# DC-SIGN Binding Contributed by an Extra N-Linked Glycosylation on Japanese Encephalitis Virus Envelope Protein Reduces the Ability of Viral Brain Invasion

**DOI:** 10.3389/fcimb.2018.00239

**Published:** 2018-07-10

**Authors:** Jian-Jong Liang, Min-Wei Chou, Yi-Ling Lin

**Affiliations:** ^1^Institute of Biomedical Sciences, Academia Sinica, Taipei, Taiwan; ^2^Genomics Research Center, Academia Sinica, Taipei, Taiwan

**Keywords:** Japanese encephalitis virus, envelope protein, glycosylation, vaccine potential, DC-SIGN

## Abstract

The major structural envelope (E) protein of Japanese encephalitis virus (JEV) facilitates cellular binding/entry and is the primary target of neutralizing antibodies. JEV E protein has one N-linked glycosylation site at N154 (G2 site), but the related dengue virus E protein has two glycosylation sites at N67 (G1 site) and N153 (G2 site). We generated three recombinant JEVs with different glycosylation patterns on the E protein. As compared with wild-type (WT) JEV with G2 glycosylation, viral growth in culture cells as well as neurovirulence and neuroinvasiveness in challenged mice were reduced when infected with the G1 mutant (E-D67N/N154A) with glycosylation shifted to G1 site, and the G0 mutant (E-N154A) with non-glycosylation. The G1G2 mutant (E-D67N), with E-glycosylation on both G1 and G2 sites, showed potent *in vitro* viral replication and *in vivo* neurovirulence, but reduced neuroinvasiveness. Furthermore, the JEV mutants with G1 glycosylation showed enhanced DC-SIGN binding, which may then lead to reduced brain invasion and explain the reason why WT JEV is devoid of this G1 site of glycosylation. Overall, the patterns of N-linked glycosylation on JEV E proteins may affect viral interaction with cellular lectins and contribute to viral replication and pathogenesis.

## Introduction

The flavivirus genus contains many important human pathogens such as Japanese encephalitis virus (JEV), dengue virus (DENV), yellow fever virus, West Nile virus (WNV), and Zika virus (ZIKV). Flaviviral virion is enveloped and contains a positive-sense, single-stranded RNA genome. The viral RNA translates a polyprotein that is proteolytically processed into structural proteins [core, precursor of membrane, and envelope (E)] and seven nonstructural proteins (NS1, NS2A, NS2B, NS3, NS4A, NS4B, and NS5) (Lindenbach et al., 2007). Embedded in the viral membrane are 180 copies of the E glycoproteins. The E glycoprotein facilitates cellular attachment, binding and entry, and is the primary target of neutralizing antibodies (Mukhopadhyay et al., 2005). The flavivirus receptors included two types of molecules, glycosaminoglycans (GAGs) and cellular proteins. Highly sulfated GAGs such as heparan sulfate serve as initial attachment factors to gather viral particles on the cell surface following interact with cellular proteins (Chen et al., 1997; Su et al., 2001; Germi et al., 2002). Several cellular proteins such as vimentin (Das et al., 2011; Liang et al., 2011), αvβ3 integrin (Chu and Ng, 2004), and heat shock protein 70 (Das et al., 2009) are putative JEV receptors.

Calcium-dependent (C-type) lectins are transmembrane proteins that primarily display on antigen-presenting cells for recognition of specific carbohydrates on glycoconjugates. Dendritic cell-specific intercellular adhesion molecule-3-grabbing nonintegrin (DC-SIGN, CD209) and its homolog DC-specific ICAM-3-grabbing nonintegrin-related protein (DC-SIGNR, L-SIGN, CD209L) can be attachment factors tethering glycosylated virions onto the cell surface (Davis et al., 2006; Zhang et al., 2014). DC-SIGN expresses on dendritic cells and a subpopulation of macrophages, whereas L-SIGN is found on the endothelial cells of placenta, liver and lymph nodes. DC-SIGN and L-SIGN were found involved in viral infection with WNV (Davis et al., 2006), DENV (Tassaneetrithep et al., 2003), and JEV (Shimojima et al., 2014; Wang et al., 2016). Furthermore, a variant in the CD209 promoter was associated with severity of dengue disease, which supports the importance of DC-SIGN in DENV infection (Sakuntabhai et al., 2005).

DENV E proteins have two potential N-linked glycosylation sites at residues N67 (G1 site) and N153 (G2 site), whereas JEV E proteins have only one N-linked glycosylation at residue N154 (G2 site). Cryo-electron microscopy (EM) study demonstrated that the carbohydrate recognition domain of DC-SIGN binds preferentially to a subset of glycans present at N67 (G1 site) on DENV particles (Pokidysheva et al., 2006). DENV lacking G1-glycosylation was able to infect mammalian cells, but production of new infectious particles was abolished (Mondotte et al., 2007). Studies of JEV also indicated the importance of glycan-lectin interaction in viral infection. For example, overexpression of DC-SIGN or L-SIGN on non-permissive cells increased JEV infection (Shimojima et al., 2014; Wang et al., 2016). Furthermore, JEV lacking N-linked glycosylation by enzyme removal or mutation of an infectious clone showed reduced interaction with DC-SIGN and loss of neuroinvasiveness in mice (Zhang et al., 2011; Wang et al., 2016). However, it is not clear whether JEV differs from DENV by using G2-glycosylation to interact with DC-SIGN as well as the molecular reasons for JEV to evolve as having only one glycan motif at the G2 site of E protein.

To address the precise role, especially the location and number of N-linked glycosylation on JEV E proteins, we generated three JEV mutants—glycosylation at N67 (G1), N67 plus N154 (G1G2), or no glycosylation (G0) on E proteins, respectively. By comparing the *in vitro* and *in vivo* properties between the wild type (WT; E-glycosylation at G2) and these mutants, we determined the contribution of N-glycans on E proteins to JEV infection.

## Materials and methods

### Ethics statement

Animal studies were conducted according to the guidelines outlined by the Council of Agriculture, Executive Yuan, Republic of China. The animal protocol was approved by the Academia Sinica Institutional Animal Care and Utilization Committee (Protocol ID 13-08-570). All surgery was performed under Isoflurane anesthesia to minimize suffering. For biosafety, we followed the “Guidelines for Research Involving Recombination DNA Molecules” issued by the Ministry of Science and Technology, Republic of China (NSC, 2004), in which JEV is classified as a BSL-2 agent.

### Cell lines and chemicals

Baby hamster kidney BHK-21 fibroblast cells (BHK-21; ATCC, CCL-10) were grown in RPMI 1640 medium containing 5% fetal bovine serum (FBS) and 2 mM L-glutamine. Human embryonic kidney 293T cells (HEK293T; ATCC, CRL-11268) and the human microglial cell line (CHME3) (Chen et al., 2012) were cultured in DMEM medium (Gibco) supplemented with 5% FBS and 2 mM L-glutamine. Human neuroblastoma SK-N-SH cells (ATCC HTB-11 cells) were grown in minimal essential medium (MEM, Eagle, Sigma-Aldrich) supplemented with 10% FBS and 2 mM L-glutamine. JEV plaques were stained with crystal violet or immunostained with anti-JEV NS3 plus alkaline phosphatase-conjugated secondary antibody (Sigma-Aldrich). For immunoplaque assay, the color was developed by adding NBT/BCIP development substrate (Promega). Mouse monoclonal antibodies specific for JEV E (PL1-365) and NS3 (J3-30) protein were described previously (Liang et al., 2011).

### Generation of recombinant JEV with site-specific glycosylation mutation

The JEV infectious clone CMV RP-9 (JEV genotype III) was used as a template to generate glycosylation-mutated viruses by single primer mutagenesis as described (Makarova et al., 2000; Liang et al., 2009) with the primer sequences for E-N154A (5′- CACTTCGGAAAACCATGGG*GCT*TATTCAGCGCAAGTTGGGG-3′) and E-D67N (5′-GTTACTGTTATCATGCTTCAGTCACT*AAT*ATCTCGACGGTGGCTCG-3′) (mutation underlined), respectively. For infectious virus production, plasmids carrying WT, or mutated JEV cDNA were transfected into BHK-21 cells, and culture supernatants were collected after incubation for 4 days. Viruses were amplified in mosquito C6/36 cells (Aedes albopictus clone C6/36; ATCC, CRL-1660) for further study (Igarashi, 1978; Chen et al., 1996).

### Virus infection and titration

For analysis of viral growth, monolayers of human neuroblastoma HTB-11 cells grown in 12-well plates were adsorbed with JEV WT or mutants for 2 h at 37°C. Unbound virus was removed by a gentle wash with serum free RPMI, then cells were cultured at 37°C. At the indicated times, culture supernatants were collected to determine infectious virus production by plaque assay on BHK-21 cells. Various virus dilutions were added onto 50% confluent BHK-21 cells and incubated at 37°C for 2 h. After adsorption, cells were washed and overlaid with 1% agarose (SeaPlaque, FMC BioProducts) containing RPMI-1640 with 2% FBS for 4 days, then fixed with 10% formaldehyde and stained with 0.5% crystal violet.

### Virus deglycosylation

To obtain extracellular E proteins, culture supernatant from JEV WT- or mutant-infected C6/36 cells was concentrated and purified by ultracentrifugation. To obtain intracellular E proteins, C6/36 cell lysates were collected. E glycoproteins were digested with N-glycosidase F (Roche) and endoglycosidase H (Roche) under reducing conditions for 16 h at 37°C according to the manufacturer's instructions.

### Mouse challenge assays

Groups of 5-week-old C57BL/6 mice were challenged by the i.p. plus i.c. route (intraperitoneally inoculated with JEV and intracerebrally injected with 30 μl PBS) or i.c. route (intracerebrally injected with 30 μl of the indicated viruses) as described previously (Hase et al., 1990; Chen et al., 1996). Groups of 5-week-old Stat-1 deficient (Stat-1^−/−^) mice (Durbin et al., 1996) were challenged by subcutaneous route with the indicated JEV E mutants. Mice survival was observed daily and the median lethal dose (LD_50_) of each virus was calculated by the Reed and Muench method (Reed and Muench, 1938). For protection test, groups of 4-week-old C57BL/6 mice were immunized with JEV E mutants by the subcutaneous route. After 2 weeks of immunization, mice were challenged with 2 × 10^5^ plaque-forming units (PFU) of a lethal JEV strain RP-9 (Chen et al., 1996) by the i.p. plus i.c. route.

### Immunoprecipitation-western blot assay

HEK293T cells infected with JEV (multiplicity of infection [MOI] = 1) for 4 h were transfected with Flag-tagged DC-SIGN by use of Lipofectamine 2000 (Invitrogen) for 24 h. Cells were rinsed with phosphate-buffered saline (PBS) and lysed with protein lysis buffer (150 mM NaCl, 50 mM Tris-HCl [pH 7.5], 1 mM EDTA, 1% NP-40, 1% Triton X-100) containing a cocktail of protease inhibitors (Roche). Cell lysates were immunoprecipitated with anti-Flag M2 affinity gel (A2220; Sigma) overnight at 4°C. The immunocomplex was washed with cold protein lysis buffer at 4°C and suspended in sample buffer with 2-mercaptoethanol. Proteins were separated with 4 to 12% NuPage (Invitrogen) and probed with anti-E antibody by western blot analysis.

### Virus binding assay

CHME3 cells with DC-SIGN or eGFP overexpression were established by transduction with lentivirus expressing DC-SIGN or eGFP. Cells were adsorbed with JEV WT or mutants at 4°C for 2 h, and then washed extensively with cold serum-free RPMI three times. Total RNA was extracted by using the RNeasy kit (QIAGEN) and analyzed by real-time RT-PCR. The cDNA was reverse transcribed from 1 μg of RNA with random hexamers by using the ThermoScript RT kit (Invitrogen). PCR involved the LightCycler FastStart DNA Master PLUS SYBR Green I kit (Roche) with the primer sequences JEV nt 10603–10619, forward (5′-AAGTTGAAGGACCAACG-3′) and JEV nt 10789–10772, reverse (5′-GCATGTTGTTGTTTCCAC-3′), or β-actin nt 811–831, forward (5′-TCCTGTGGCATCCACGAAACT-3′) plus β-actin nt 1125–1105, reverse (5′-GAAGCATTTGCGGTGGACGAT-3′). For relative quantification, JEV RNA was measured relative to actin RNA in each sample and the concentration was calculated from a standard curve. Melting curves were used to verify the specificities of products.

### Statistical analysis

Data are presented as mean ± standard deviation (*SD*). Statistical analysis was performed using one-way analysis of variance (ANOVA) with Prism (GraphPad Software) and two-tailed Student *t-*test. Determination of the median survival time (T_50_) and *P*-values by the log-rank test, involved use of Prism (GraphPad Software).

## Results

### *In vitro* properties of E glycosylation-mutated JEVs

**Figure 1:**
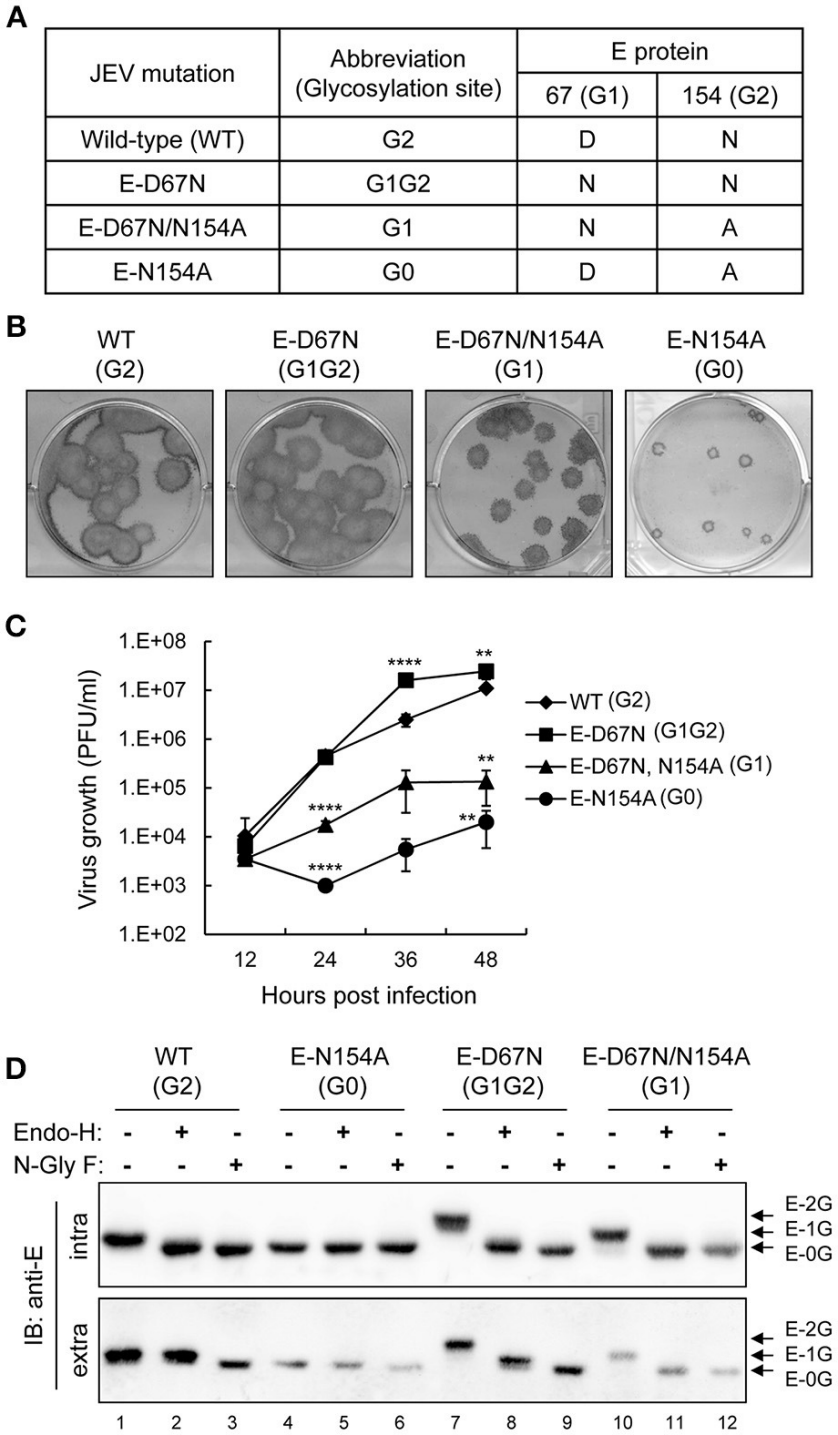
*In vitro* properties of E glycosylation-mutated JEVs. **(A)** JEV wild-type (WT) and E protein N-linked glycosylation mutants used in this study. **(B)** Plaque morphology of JEV WT and mutants on BHK-21 cells immunostained with anti-JEV NS3 antibody. **(C)** Growth curves of JEV WT and mutants in human neuroblastoma HTB-11 cells. Culture supernatants were collected at the indicated times and titrated by plaque forming assays on BHK-21 cells. Data are mean ± *SD* (*n* = 3). The virus titers were compared to WT (G2) at the same time point using one-way ANOVA with Bonferroni multiple comparisons test. ^**^P < 0.01; ^****^P < 0.0001. **(D)** Western blot analysis of cell lysates and culture supernatants from C6/36 cells infected with the indicated JEVs and treated with glycosidase N-GlyF, Endo-H or buffer only, then immunoblotted with anti-E antibody. The positions of E proteins with two, one and no glycosylation are marked as E-2G, E-1G, and E-0G, respectively.

To understand whether the position and number of N-linked glycosylation on JEV E protein affected its biological properties, we generated three recombinant viruses with mutated E proteins (Figure 1A). The WT E protein of JEV had one N-linked glycosylation at residue N154 (G2 site). The E-D67N mutant with one extra N-linked glycosylation site at residue N67 (G1 site) had two glycan motifs (G1G2), similarly to DENV. The E-D67N/N154A double mutant had a changed N-linked glycan motif from the G2 to G1 site, and the E-N154A mutation produced the G0 mutant with no N-linked glycosylation. Plaque morphology of the G1G2 mutant and the WT JEV was similar, whereas the G1 mutant had slightly smaller plaque, and the G0 even smaller plaque (Figure 1B). The pattern of viral growth was similar to that for plaque morphology: WT (G2) ≈ G1G2 > G1 > G0 (Figure 1C).

Glycosylation patterns of JEV E proteins were assayed by using two enzymes, N-glycosidase F (N-GlyF), which removes all N-linked carbohydrates, and endoglycosidase H (Endo-H), which only removes high mannose and some hybrid types of N-linked carbohydrates. G0 has no N-linked carbohydrates, so the gel mobility of E proteins was similar with or without enzyme treatment (Figure 1D, lanes 4–6), whereas gel mobility was increased with enzyme treatment for the WT (lanes 1–3), G1G2 (lanes 7–9), and G1 (lanes 10–12). For intracellular E proteins, gel mobility was similar with N-GlyF or Endo-H treatment for all JEVs (Figure 1D, upper panel), which suggests high-mannose glycans for both G1 and G2 sites of the intracellular E protein. However, mobility differences were noted for extracellular E proteins of WT (G2) and G1G2, but not G0 and G1, treated with N-GlyF or Endo-H (Figure 1D, lower panel), indicating that G2 but not G1 glycosylation was further modified from high-mannose to a complexed form during transport through the Golgi apparatus. Thus, different from the intracellular E proteins with high-mannose glycans at both G1 and G2 sites, for the extracellular E proteins, G1 contained high-mannose glycans, but G2 contained complexed forms of glycans.

### *In vivo* properties of E glycosylation-mutated JEVs

**Figure 2:**
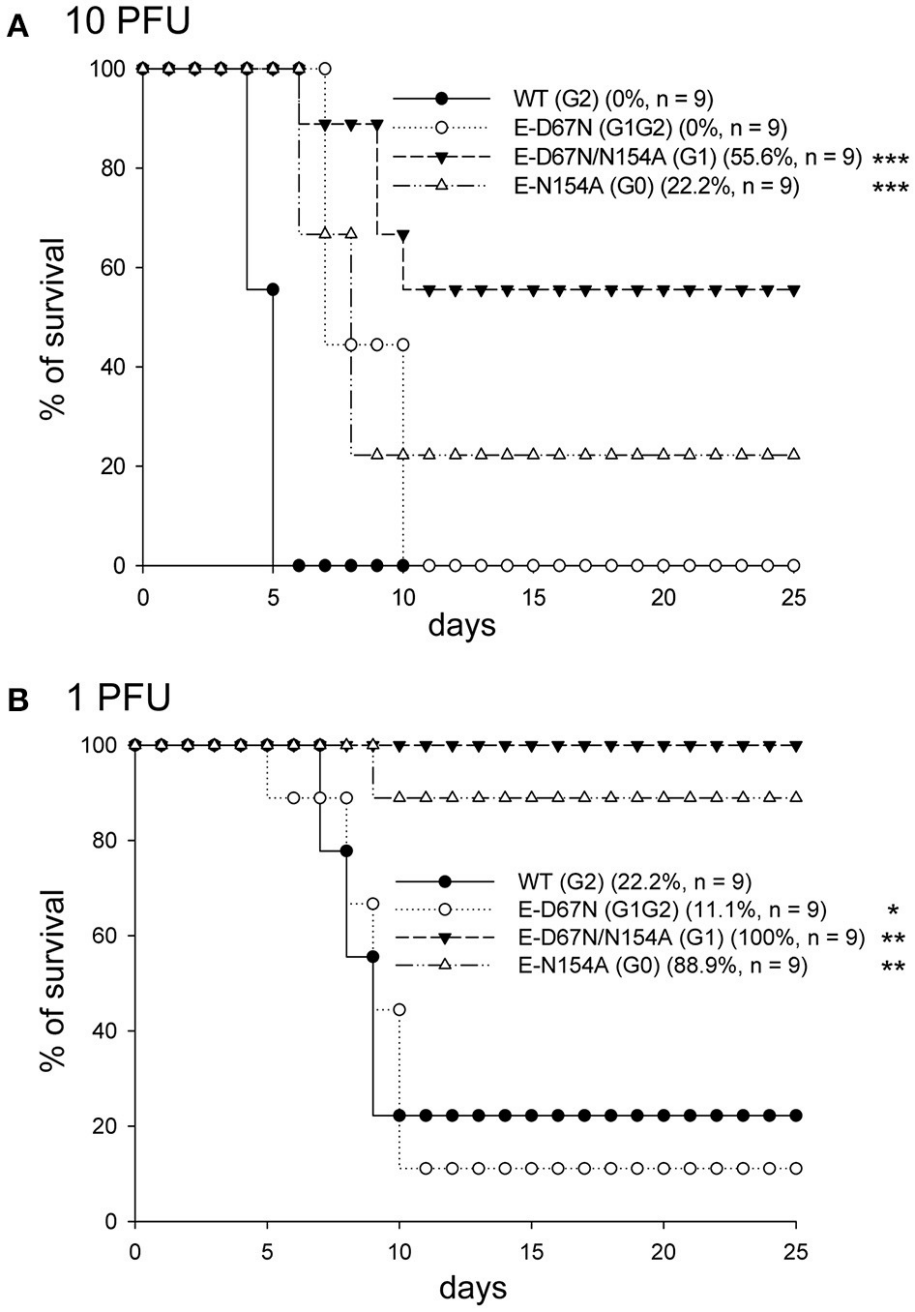
Neurovirulence of JEV WT and mutants. C57BL/6 mice were intracerebrally (ic) inoculated with the indicated JEVs at 10 plaque-forming units (PFU) **(A)** or 1 PFU **(B)** per mouse and observed daily. The survival rates and number of mice in each group are shown. Prism software with the log rank test was used to compare survival curves with WT. ^*^P < 0.05; ^**^P < 0.01; ^***^P < 0.001.

**Figure 3:**
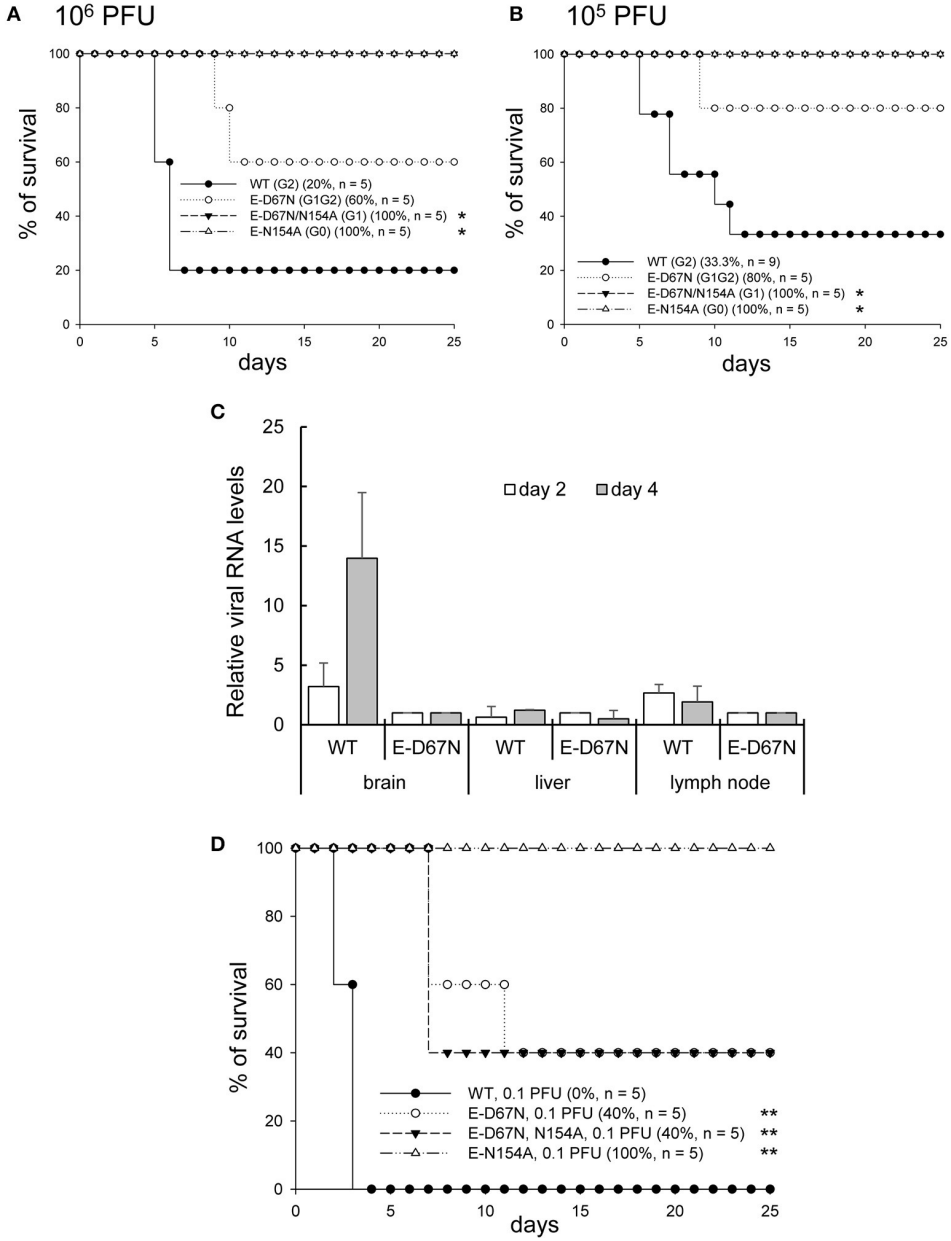
Neuroinvasiveness of JEV WT and mutants. C57BL/6 mice were intraperitoneally (ip) inoculated with the indicated JEVs at 10^6^ PFU **(A)** or 10^5^ PFU **(B)** per mouse and simultaneously injected intracerebrally (ic) with 30 μl PBS (ip plus ic route). The survival of mice was observed daily. Prism software with the log rank test was used to compare survival curves with WT. ^*^P < 0.05. **(C)** RT-qPCR of relative JEV RNA levels in brain, liver, and lymph node tissues of mice inoculated with WT or E-D67N mutant (10^6^ PFU/mouse) (*n* = 3). **(D)** 5-week-old Stat-1 knockout mice were subcutaneously infected with JEV E mutants (0.1 PFU/mouse). The number of mice (*n*) in each experimental group is shown in the legend to each panel. Prism software with the log rank test was used to compare survival curves with WT. ^**^P < 0.01.

To study the *in vivo* effect of JEV E glycosylation, we determined the neurovirulence and neuroinvasiveness of the WT and mutants in mice with intracranial (challenged with 10 and 1 PFU/mouse) and peripheral injection (challenged with 10^6^ and 10^5^ PFU/mouse), respectively. G1 and G0 mutants showed reduced neurovirulence (LD_50_ 12.8 and 4.2 PFU, respectively) as compared with the G1G2 mutant and WT JEV (LD_50_ 0.36 and 0.44 PFU, respectively) (Figure 2 and Table 1). Longer median survival time (T_50_) was also noted for the attenuated G1 and G0 mutants (Table 1). For neuroinvasiveness, all mice survived the challenge of G1 and G0 mutants, indicating reduced neuroinvasiveness for these two mutants (Figure 3A,B). Furthermore, lower neuroinvasiveness was noted for G1G2 with higher LD_50_ (>10^6^ PFU for G1G2 vs. ~10^4^ PFU for WT) and longer T_50_ (>25 days for G1G2 vs. 7 and 11 days for the two challenge doses for WT) Table 1). An increase of viral RNA in the brain tissues of mice challenged with WT JEV, but not much for G1G2 mutant, also indicated a weakening of G1G2 to cross the blood brain barrier (Figure 3C). To further support the data of neuroinvasiveness, we challenged Stat-1^−/−^ mice via subcutaneous route. Lower neuroinvasiveness was noted for G1G2 with higher survival rate (40% for G1G2 vs. 0% for WT) (Figure 3D) and longer T_50_ (12 days for G1G2 vs. 4 days for WT) (Table 1). Thus, the JEV E-D67N (G1G2) mutant with an extra N-linked glycosylation at JEV E protein N67 did not show impaired neurovirulence but rather impeded ability to invade the central nervous system.

**Table 1:** The neurovirulence and neuroinvasiveness of JEV WT and mutants in C57BL/6^a^ and Stat1^−/−b^ mice.

	**Neurovirulence**			**Neuroinvasiveness**			
	**LD_50_^a^ (PFU)**	**T_50_^a^ (10 PFU)**	**T_50_^a^ (1 PFU)**	**LD_50_^a^ (PFU)**	**T_50_^a^ (10^6^ PFU)**	**T_50_^a^ (10^5^ PFU)**	**T_50_^b^ (0.1 PFU)**
WT (G2)	0.44	6	10	~10^4^	7	11	4
E-D67N (G1G2)	0.36	8	10	>10^6^	>25	>25	12
E-D67N/N154A (G1)	12.8	>25	>25	>10^6^	>25	>25	8
E-N154A (G0)	4.2	9	>25	>10^6^	>25	>25	>25

*LD_50_, median lethal dose; T_50_, median survival time; PFU, plaque forming unit*.

### Vaccine potential of JEV E-glycosylation mutants

**Figure 4:**
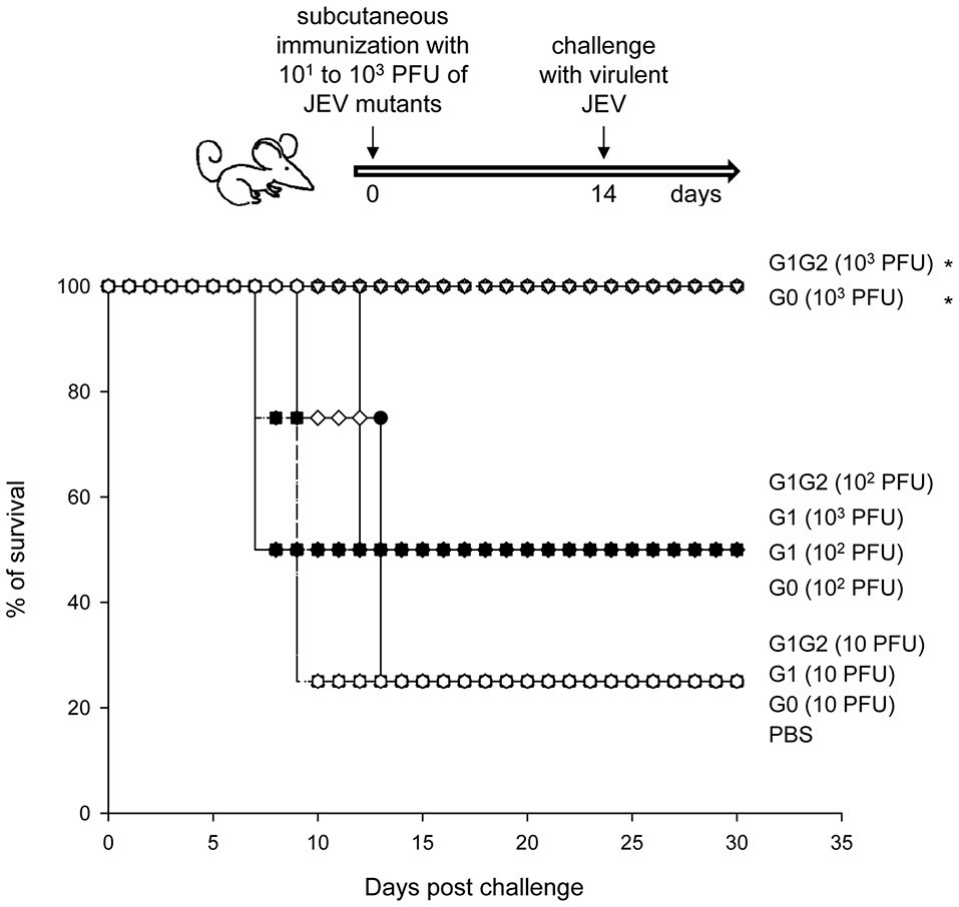
Vaccine potential of JEV E-glycosylation mutants. Four-week-old C57BL/6 mice (*n* = 4 per group) were subcutaneously injected with JEV E-glycosylation mutants at 10^1^, 10^2^, or 10^3^ PFU per mouse. Two weeks after immunization, mice were challenged with 2 × 10^5^ PFU of the JEV WT RP-9 strain by the ip plus ic route. Mouse survival was observed daily. Prism software with the log rank test was used to compare survival curves. ^*^P < 0.05.

To determine whether these E-glycosylation-mutated JEVs induced protective immunity, we vaccinated C57BL/6 mice once by subcutaneous injection with various doses of JEV mutants (10^1^, 10^2^ and 10^3^ PFU per mouse), then challenged with a virulent JEV strain 2 weeks later. A single vaccination with 10^3^ PFU of G1G2 or G0 completely protected the mice against JEV challenge, whereas degrees of mortality were noted in groups vaccinated with lower doses of JEVs or the PBS control (Figure 4). Because the G1G2 mutant replicated to much higher titers than the G0 mutant (Figure 1C), the G1G2 mutant had the growth advantage and can be further considered as a JEV vaccine candidate.

### Glycosylation at N67 of JEV E protein enhanced binding with DC-SIGN

**Figure 5:**
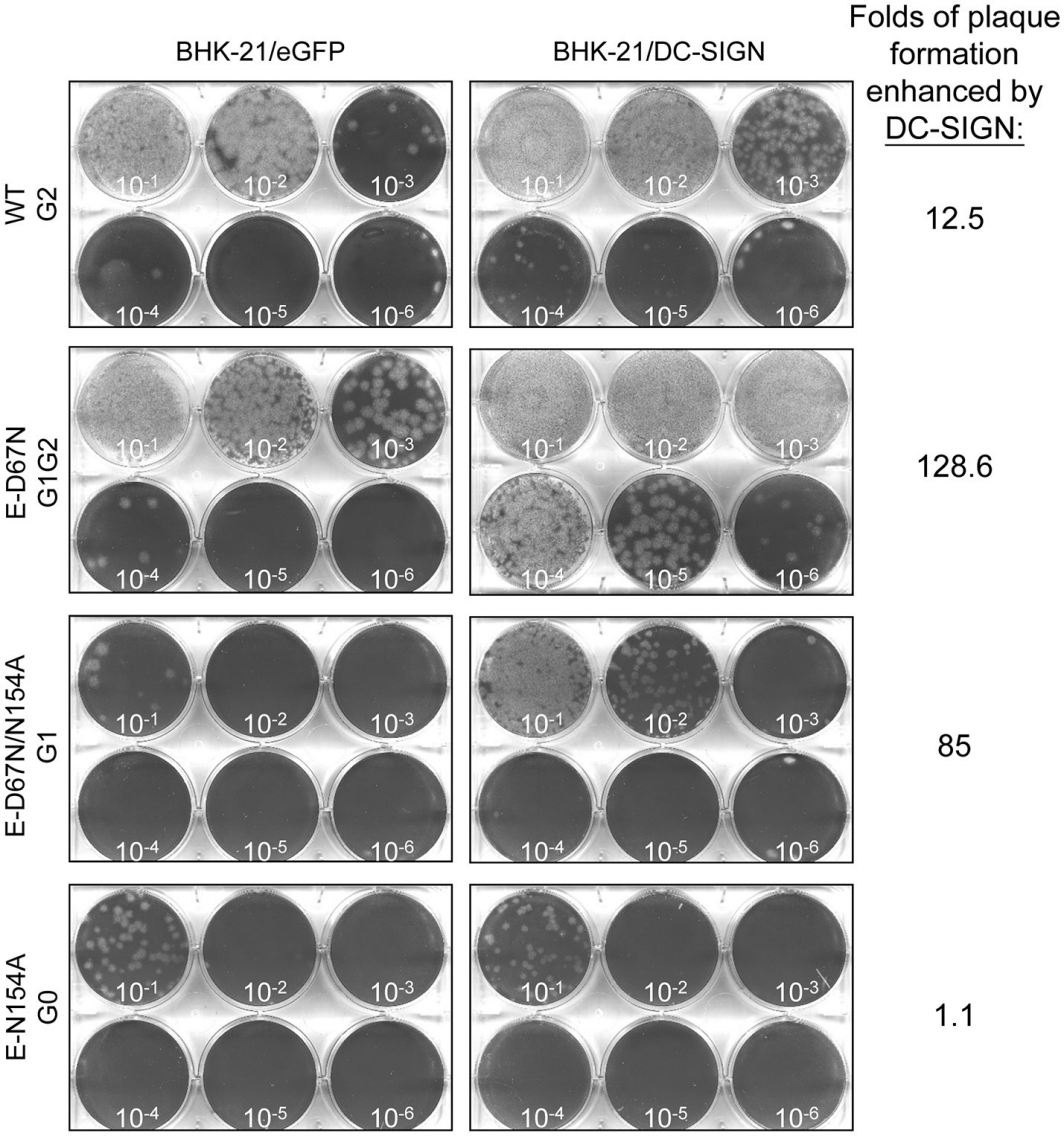
Plaque-forming ability of JEV WT and mutants in cells with or without DC-SIGN. BHK-21 cells overexpressing eGFP or DC-SIGN were infected with serially diluted JEV WT or mutants, then overlaid with agarose-containing medium for plaque formation. After incubation for 4 days, cells were fixed with formalin and stained with crystal violet. The number of plaques formed were counted; folds of plaque formation enhanced by DC-SIGN as compared with eGFP control are shown.

To understand the molecular mechanism contributing to the reduced neuroinvasiveness of the G1G2 mutant, we tested whether the extra N-linked glycosylation at the G1 site affected JEV's interaction with cell-surface lectins such as DC-SIGN. By using the same aliquots of serially diluted JEV WT and mutants to form plaques on BHK-21 cells overexpressing DC-SIGN or the eGFP control, we compared the infectivity of JEV on cells with or without DC-SIGN expression. Increased plaque formation in DC-SIGN-overexpressing cells was noted for the three JEVs with glycosylated E proteins (WT, G1G2, and G1), but not for the glycosylation-null G0 mutant, as compared to the GFP control cells (Figure 5). Furthermore, the plaque-formation enhancing effects differed among these three JEVs: G1G2 (128.6-fold) > G1 (85-fold) > WT G2 (12.5-fold). Thus, the glycan motif on G1 site might be more important than that on G2 to enhance JEV E protein interacting with DC-SIGN.

**Figure 6:**
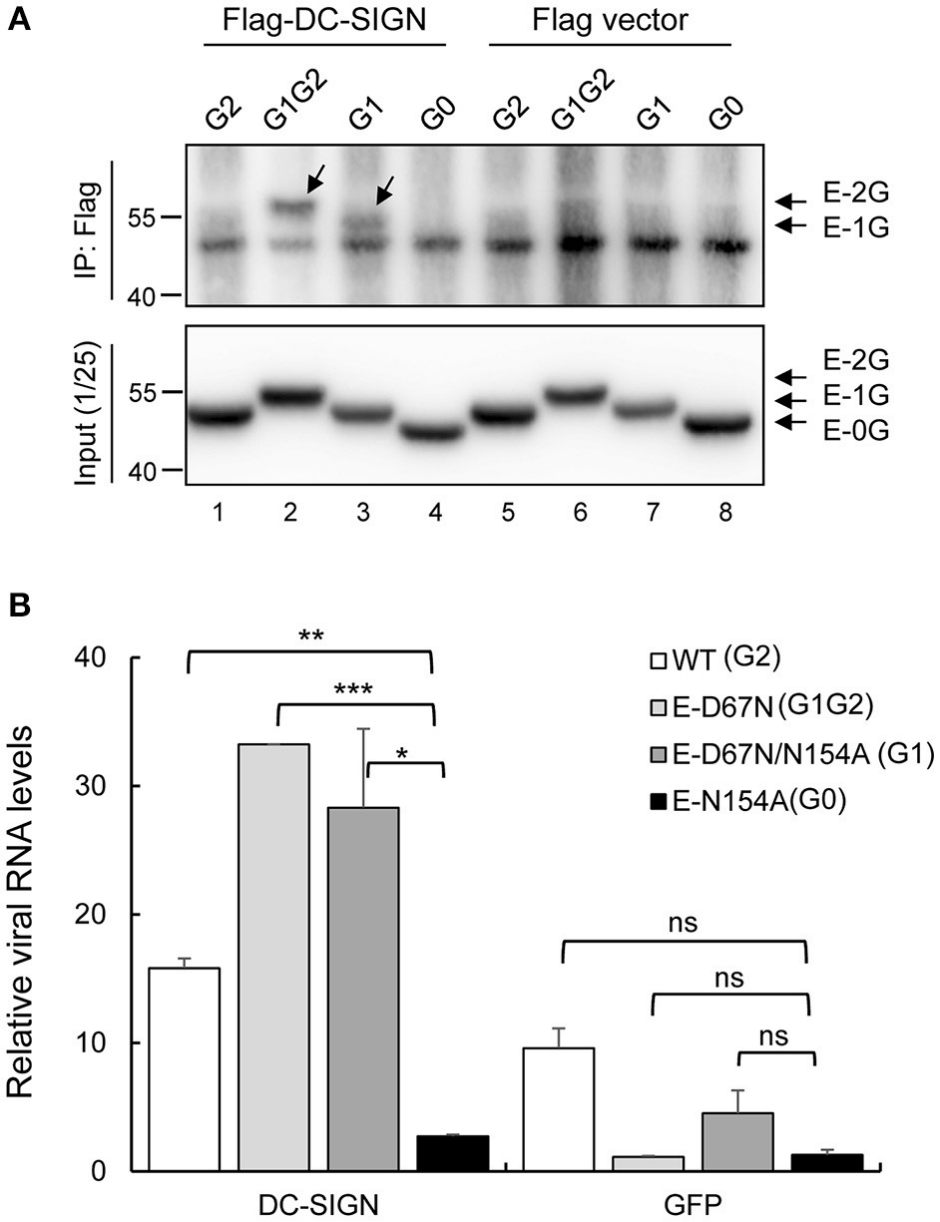
Interaction of JEV WT and mutants with DC-SIGN. **(A)** HEK293T cells were infected with JEV for 4 h, then transfected with Flag-DC-SIGN or control vector for 24 h. Cell lysates were immunoprecipitated with anti-Flag conjugated beads and then probed with anti-E antibody by western blot analysis. **(B)** Human CHME3 cells overexpressing DC-SIGN or GFP were adsorbed with JEV WT or mutants at 4°C for 2 h, then underwent RNA extraction and real-time RT-PCR. The amount of cell-associated virus was quantified as the relative JEV copy number normalized to that of actin. Data are mean ± *SD* from three independent samples. The RNA level of the indicated groups were compared using Student's *t-*test. ^*^P < 0.05; ^**^P < 0.01; ^***^P < 0.001; ns, not significant.

We then tested the ability of WT and mutant JEVs to bind with DC-SIGN by using two binding assays. The first was immunoprecipitation-western blot assay of HEK293T cells infected with JEVs and co-transfected with Flag-tagged DC-SIGN or vector control by using anti-Flag antibody-conjugated beads and anti-JEV E antibody. Interaction of DC-SIGN with JEV E protein was readily seen in G1G2 and G1 mutants but not much in G2 and G0 mutants (Figure 6A). Next, we measured the amount of viral binding at 4°C for 2 h by detecting viral RNA with real-time RT-PCR on CHME3 cells overexpressing DC-SIGN or GFP control. The amount of cell surface-associated virus was quantified as relative JEV copy number normalized to that of actin. DC-SIGN-overexpressing cells showed greater viral binding as compared with the GFP control, especially for JEVs with E glycosylation. Furthermore, the amount of G1G2 and G1 adsorbed onto DC-SIGN-expressing cells was even higher than that of WT G2 and G0 (Figure 6B). In accordance with the infectivity assay (Figure 5), JEV E proteins with G1 glycosylation in G1G2 and G1 mutants showed enhanced binding with DC-SIGN.

## Discussion

Mature flaviviral particles have 180 copies of E glycoproteins and M proteins embedded in the viral membrane (Kuhn et al., 2002). Cryo-EM studies of flaviviruses demonstrate a common organization pattern with remarkable structural similarity. The most notable difference between flaviviruses is in the region near the glycosylation site (Sirohi and Kuhn, 2017). The location and number of glycosylation sites varies across flaviviruses. Four serotypes of DENV have two potential N-linked glycosylation motifs on E protein residue N67 and N153, but neurotropic flaviviruses such as JEV, WNV and ZIKV have only a single N-linked glycosylation motif on N154. In this study, we added an extra glycosylation site to the WT JEV, so the resulting G1G2 mutant had two glycosylation motifs on its E protein, similarly to DENV. G1G2 showed comparable *in vivo* neurovirulence as the WT JEV, but its neuroinvasiveness was reduced. Thus, the neurotropic flaviviral E proteins with only one glycosylation at N153 might have a selection advantage for crossing the blood-brain barrier during evolution. This finding also supports that glycans on flaviviral E proteins play important roles in viral infection and affect viral tropism.

Glycan diversity on the virion surface should be related to differential usage of lectin receptors by these flaviviruses (Sirohi and Kuhn, 2017). In reporter viral particles pseudotyped with WNV E proteins, adding an extra N-linked glycosylation at N67 of WNV E protein increased the infection of DC-SIGN-overexpressing cells (Davis et al., 2006). Here, we used an infectious clone to create recombinant JEVs that differed only in the E-glycosylation pattern. JEV mutants with single or extra glycosylation at N67 of E proteins (G1 and G1G2) showed increased binding to DC-SIGN as compared with the WT (G2), which suggests that G1-glycosylation on N67 is important for DC-SIGN binding. Because DC-SIGN is known to bind with high-mannose-type glycans (van Kooyk and Geijtenbeek, 2003; Wang et al., 2016), we analyzed the glycosylation patterns of these JEV E mutants (Figure 1D). The G1 glycans were sensitive to endo-H cleavage, which indicates a high-mannose type for both intracellular and extracellular E proteins. However, the G2 glycans on intracellular and extracellular E proteins showed different endo-H sensitivity, indicating a high-mannose form for intracellular E protein and a complexed form for extracellular E protein. Thus, the high mannose-type glycans of G1 likely contributed to interacting with DC-SIGN. However, the WT JEV, with a single glycosylation at G2, still bound to DC-SIGN as compared with the glycosylation-null G0 mutant (Figure 6B). Whether the G2-site glycans still contribute to DC-SIGN binding or whether other glycosylation such as that on precursor of membrane (prM) protein caused the binding for WT JEV remains to be studied.

Single amino acid replacements in structural proteins have been found to affect receptor usage, tropism and pathogenesis for many viruses (Baranowski et al., 2001). Flaviviruses used highly sulfated GAGs such as heparan sulfate to initiate cell binding (Chen et al., 1997; Su et al., 2001; Germi et al., 2002), whereas high GAG binding ability often results in flaviviral attenuation (Lee and Lobigs, 2000; Lee et al., 2004). Glutamate to lysine mutation of flaviviral E proteins at residue 138 enhanced GAG binding *in vitro*, have an attenuation phenotype in animals and lead to vaccine potential (Lee et al., 2004). Glycosylation mutants of flaviviruses have also been considered to have vaccine potential (Kim et al., 2008; Zhang et al., 2011). The viral properties of WNV devoid of glycosylation were attenuated and induced protective immunity (Hanna et al., 2005; Moudy et al., 2009), but the virus did not grow to high titers (Whiteman et al., 2010). Similarly, JEV G0 mutant without E-glycosylation could also confer protective immunity (Figure 4). Furthermore, the G1G2 mutant of JEV showed similar protection ability as G0, and the growth advantage of G1G2 suggests a more attractive vaccine candidate. Furthermore, besides its role in ligand binding, DC-SIGN also rapidly internalizes and directs its cargo into the endo-lysosomal pathway, which results in major histocompatibility complex class II presentation (van Kooyk et al., 2013). The enhanced DC-SIGN binding of G1-glycosylated JEV might have the vaccine potential by boosting glycan-mediated antigen presentation for specific immune responses.

JEV activity has been reported in most of Southeast Asia (Fan et al., 2017). Nearly 70,000 cases of JE infections occur worldwide annually; 75% of these JE infections involve children age 0 to 14 years (Campbell et al., 2011; WHO, 2016). Most JE infections in humans cause an acute non-specific febrile illness, whereas children and older people may show mild infection to severe encephalitis. No curative therapy or drugs exist for JE infection and the treatment is only symptomatic. Available vaccines against JEV include both inactivated whole virus and live attenuated vaccines. The use of inactivated virions in national vaccination programs is limited by high cost, risk of allergic reaction, and the need for multiple doses. The SA 14-14-2 live attenuated vaccine is widely used in China and increasingly in other Asian countries. However, vaccine attenuation stability remains a concern for its expanded application. The approach of adding glycosylation at the G1 site of E protein might increase vaccine safety without hampering efficacy. Our study also provides a comprehensive knowledge of the key host molecules and pathways involved in virus infection, which is crucial to understand virus host interplays and to develop novel vaccines.

## Author contributions

J-JL and Y-LL designed research, analyzed data, and wrote the paper. J-JL and M-WC performed research and animal study. M-WC provided technical assistance.

### Conflict of interest statement

The authors declare that the research was conducted in the absence of any commercial or financial relationships that could be construed as a potential conflict of interest.

## References

[B1] Baranowski E. Ruiz-Jarabo C. M. Domingo E. (2001). Evolution of cell recognition by viruses. Science 292, 1102–1105. 10.1126/science.1058613 11352064

[B2] Campbell G. L. Hills S. L. Fischer M. Jacobson J. A. Hoke C. H. Hombach J. M.. (2011). Estimated global incidence of Japanese encephalitis: a systematic review. Bull. World Health Organ 89, 766–774, 774A−774E. 10.2471/BLT.10.085233 22084515 PMC3209971

[B3] Chen L. K. Lin Y. L. Liao C. L. Lin C. G. Huang Y. L. Yeh C. T.. (1996). Generation and characterization of organ-tropism mutants of Japanese encephalitis virus *in vivo* and *in vitro*. Virology 223, 79–88. 10.1006/viro.1996.0457 8806542

[B4] Chen S. T. Liu R. S. Wu M. F. Lin Y. L. Chen S. Y. Tan D. T.. (2012). CLEC5A regulates Japanese encephalitis virus-induced neuroinflammation and lethality. PLoS Pathog. 8:e1002655. 10.1371/journal.ppat.1002655 22536153 PMC3334897

[B5] Chen Y. Maguire T. Hileman R. E. Fromm J. R. Esko J. D. Linhardt R. J.. (1997). Dengue virus infectivity depends on envelope protein binding to target cell heparan sulfate. Nat. Med. 3, 866–871. 10.1038/nm0897-866 9256277

[B6] Chu J. J. Ng M. L. (2004). Interaction of West Nile virus with αvβ3 integrin mediates virus entry into cells. J. Biol. Chem. 279, 54533–54541. 10.1074/jbc.M410208200 15475343

[B7] Das S. Laxminarayana S. V. Chandra N. Ravi V. Desai A. (2009). Heat shock protein 70 on Neuro2a cells is a putative receptor for Japanese encephalitis virus. Virology 385, 47–57. 10.1016/j.virol.2008.10.025 19068261

[B8] Das S. Ravi V. Desai A. (2011). Japanese encephalitis virus interacts with vimentin to facilitate its entry into porcine kidney cell line. Virus Res. 160, 404–408. 10.1016/j.virusres.2011.06.001 21798293

[B9] Davis C. W. Mattei L. M. Nguyen H. Y. Ansarah-Sobrinho C. Doms R. W. Pierson T. C. (2006). The location of asparagine-linked glycans on West Nile virions controls their interactions with CD209 (dendritic cell-specific ICAM-3 grabbing nonintegrin). J. Biol. Chem. 281, 37183–37194. 10.1074/jbc.M605429200 17001080

[B10] Durbin J. E. Hackenmiller R. Simon M. C. Levy D. E. (1996). Targeted disruption of the mouse Stat1 gene results in compromised innate immunity to viral disease. Cell 84, 443–450. 10.1016/S0092-8674(00)81289-1 8608598

[B11] Fan Y. C. Lin J. W. Liao S. Y. Chen J. M. Chen Y. Y. Chiu H. C.. (2017). Virulence of Japanese encephalitis virus genotypes I and III, Taiwan. Emerg. Infect. Dis. 23, 1883–1886. 10.3201/eid2311.161443 29048288 PMC5652437

[B12] Germi R. Crance J. M. Garin D. Guimet J. Lortat-Jacob H. Ruigrok R. W.. (2002). Heparan sulfate-mediated binding of infectious dengue virus type 2 and yellow fever virus. Virology 292, 162–168. 10.1006/viro.2001.1232 11878919

[B13] Hanna S. L. Pierson T. C. Sanchez M. D. Ahmed A. A. Murtadha M. M. Doms R. W. (2005). N-linked glycosylation of west nile virus envelope proteins influences particle assembly and infectivity. J. Virol. 79, 13262–13274. 10.1128/JVI.79.21.13262-13274.2005 16227249 PMC1262570

[B14] Hase T. Dubois D. R. Summers P. L. (1990). Comparative study of mouse brains infected with Japanese encephalitis virus by intracerebral or intraperitoneal inoculation. Int. J. Exp. Pathol. 71, 857–869. 2177623 PMC2002376

[B15] Igarashi A. (1978). Isolation of a Singh's *Aedes albopictus* cell clone sensitive to Dengue and Chikungunya viruses. J. Gen. Virol. 40, 531–544. 10.1099/0022-1317-40-3-531 690610

[B16] Kim J. M. Yun S. I. Song B. H. Hahn Y. S. Lee C. H. Oh H. W.. (2008). A single N-linked glycosylation site in the Japanese encephalitis virus prM protein is critical for cell type-specific prM protein biogenesis, virus particle release, and pathogenicity in mice. J. Virol. 82, 7846–7862. 10.1128/JVI.00789-08 18524814 PMC2519568

[B17] Kuhn R. J. Zhang W. Rossmann M. G. Pletnev S. V. Corver J. Lenches E.. (2002). Structure of dengue virus: implications for flavivirus organization, maturation, and fusion. Cell 108, 717–725. 10.1016/S0092-8674(02)00660-8 11893341 PMC4152842

[B18] Lee E. Lobigs M. (2000). Substitutions at the putative receptor-binding site of an encephalitic flavivirus alter virulence and host cell tropism and reveal a role for glycosaminoglycans in entry. J. Virol. 74, 8867–8875. 10.1128/JVI.74.19.8867-8875.2000 10982329 PMC102081

[B19] Lee E. Hall R. A. Lobigs M. (2004). Common E protein determinants for attenuation of glycosaminoglycan-binding variants of Japanese encephalitis and West Nile viruses. J. Virol. 78, 8271–8280. 10.1128/JVI.78.15.8271-8280.2004 15254199 PMC446099

[B20] Liang J. J. Liao C. L. Liao J. T. Lee Y. L. Lin Y. L. (2009). A Japanese encephalitis virus vaccine candidate strain is attenuated by decreasing its interferon antagonistic ability. Vaccine 27, 2746–2754. 10.1016/j.vaccine.2009.03.007 19366580

[B21] Liang J. J. Yu C. Y. Liao C. L. Lin Y. L. (2011). Vimentin binding is critical for infection by the virulent strain of Japanese encephalitis virus. Cell. Microbiol. 13, 1358–1370. 10.1111/j.1462-5822.2011.01624.x 21707907

[B22] Lindenbach B. D. Thiel H.-J. Rice C. M. (2007). Flaviviridae: the viruses and their replication, in Fields Virology, 5th Edn., eds Knipe D. M. Howley P. M. (Philadelphia, PA: Lippincott Williams & Wilkins, a Wolters Kluwer Business), 1101–1152.

[B23] Makarova O. Kamberov E. Margolis B. (2000). Generation of deletion and point mutations with one primer in a single cloning step. Biotechniques 29, 970–972. 11084856 10.2144/00295bm08

[B24] Mondotte J. A. Lozach P. Y. Amara A. Gamarnik A. V. (2007). Essential role of dengue virus envelope protein N glycosylation at asparagine-67 during viral propagation. J. Virol. 81, 7136–7148. 10.1128/JVI.00116-07 17459925 PMC1933273

[B25] Moudy R. M. Zhang B. Shi P. Y. Kramer L. D. (2009). West Nile virus envelope protein glycosylation is required for efficient viral transmission by Culex vectors. Virology 387, 222–228. 10.1016/j.virol.2009.01.038 19249803 PMC2742948

[B26] Mukhopadhyay S. Kuhn R. J. Rossmann M. G. (2005). A structural perspective of the *flavivirus* life cycle. Nat. Rev. Microbiol. 3, 13–22. 10.1038/nrmicro1067 15608696

[B27] NSC (2004). 基因重組實驗守則 “Guidelines of for research involving recombinant DNA molecules,” Appendix II, Risk Factor group 2 (Taipei), 51.

[B28] Pokidysheva E. Zhang Y. Battisti A. J. Bator-Kelly C. M. Chipman P. R. Xiao C.. (2006). Cryo-EM reconstruction of dengue virus in complex with the carbohydrate recognition domain of DC-SIGN. Cell 124, 485–493. 10.1016/j.cell.2005.11.042 16469696

[B29] Reed L. J. Muench H. (1938). A simple method of estimating fifty per cent endpoints12. Am. J. Epidemiol. 27, 493–497. 10.1093/oxfordjournals.aje.a118408

[B30] Sakuntabhai A. Turbpaiboon C. Casadémont I. Chuansumrit A. Lowhnoo T. Kajaste-Rudnitski A.. (2005). A variant in the CD209 promoter is associated with severity of dengue disease. Nat. Genet. 37, 507–513. 10.1038/ng1550 15838506 PMC7096904

[B31] Shimojima M. Takenouchi A. Shimoda H. Kimura N. Maeda K. (2014). Distinct usage of three C-type lectins by Japanese encephalitis virus: DC-SIGN, DC-SIGNR, and LSECtin. Arch. Virol. 159, 2023–2031. 10.1007/s00705-014-2042-2 24623090 PMC7087284

[B32] Sirohi D. Kuhn R. J. (2017). Zika virus structure, maturation, and receptors. J. Infect. Dis. 216, S935–S944. 10.1093/infdis/jix515 29267925 PMC5853281

[B33] Su C. M. Liao C. L. Lee Y. L. Lin Y. L. (2001). Highly sulfated forms of heparin sulfate are involved in Japanese encephalitis virus infection. Virology 286, 206–215. 10.1006/viro.2001.0986 11448173

[B34] Tassaneetrithep B. Burgess T. H. Granelli-Piperno A. Trumpfheller C. Finke J. Sun W.. (2003). DC-SIGN (CD209) mediates dengue virus infection of human dendritic cells. J. Exp. Med. 197, 823–829. 10.1084/jem.20021840 12682107 PMC2193896

[B35] van Kooyk Y. Geijtenbeek T. B. (2003). DC-SIGN: escape mechanism for pathogens. Nat. Rev. Immunol. 3, 697–709. 10.1038/nri1182 12949494

[B36] van Kooyk Y. Unger W. W. Fehres C. M. Kalay H. García-Vallejo J. J. (2013). Glycan-based DC-SIGN targeting vaccines to enhance antigen cross-presentation. Mol. Immunol. 55, 143–145. 10.1016/j.molimm.2012.10.031 23158834

[B37] Wang P. Hu K. Luo S. Zhang M. Deng X. Li C.. (2016). DC-SIGN as an attachment factor mediates Japanese encephalitis virus infection of human dendritic cells via interaction with a single high-mannose residue of viral E glycoprotein. Virology 488, 108–119. 10.1016/j.virol.2015.11.006 26629951

[B38] Whiteman M. C. Li L. Wicker J. A. Kinney R. M. Huang C. Beasley D. W.. (2010). Development and characterization of non-glycosylated E and NS1 mutant viruses as a potential candidate vaccine for West Nile virus. Vaccine 28, 1075–1083. 10.1016/j.vaccine.2009.10.112 19896447

[B39] WHO (2016). Japanese encephalitis vaccines: WHO position paper, February 2015–Recommendations. Vaccine 34, 302–303. 10.1016/j.vaccine.2015.07.057 26232543

[B40] Zhang F. Ren S. Zuo Y. (2014). DC-SIGN, DC-SIGNR and LSECtin: C-type lectins for infection. Int. Rev. Immunol. 33, 54–66. 10.3109/08830185.2013.834897 24156700

[B41] Zhang Y. Chen P. Cao R. Gu J. (2011). Mutation of putative N-linked glycosylation sites in Japanese encephalitis virus premembrane and envelope proteins enhances humoral immunity in BALB/C mice after DNA vaccination. Virol. J. 8:138. 10.1186/1743-422X-8-138 21439032 PMC3088903

